# Multimorbidity and tooth loss: the Brazilian National Health Survey, 2019

**DOI:** 10.1186/s12889-021-12392-2

**Published:** 2021-12-20

**Authors:** Rafael Aiello Bomfim, Andreia Morales Cascaes, Cesar de Oliveira

**Affiliations:** 1 School of Dentistry, Federal University of Mato Grosso do Sul, Campo Grande, Brazil; 2 Department of Public Health, Federal University of Santa Catarina, Florianópolis, Brazil; 3 Department of Epidemiology & Public Health, University College of London, London, UK

**Keywords:** Multimorbidity, Tooth loss, Functional dentition, Severe tooth loss, Ageing, Oral health

## Abstract

**Background:**

Little is known about the presence of two or more chronic conditions (multimorbidity) on tooth loss between adults and older adults**.** Understanding the mechanisms of multimorbidity on tooth loss is essential to inform policy development. This study aims to investigate the association between multimorbidity and severity of tooth loss in Brazilian adults and older adults.

**Methods:**

We analysed data from a nationally representative sample of 88,531 Brazilian individuals aged 18 and over who participated in the 2019 Brazilian Health Survey. Tooth loss was the outcome by two different classifications: functional dentition (lost 1–12 teeth) and severe tooth loss (lost 23–32 teeth). The presence of multimorbidity was the main exposure and based on 13 self-reported doctor-diagnosed chronic diseases that were further categorised into two groups, i.e., ≥2 or ≥ 3 comorbidities. Sociodemographic covariates included sex, age, race, income, level of education and tobacco smoking and geographic region of residency. Multivariate logistic regression models estimated the OR (Odds Ratios) and 95%CI of the associations between multimorbidity and tooth loss.

**Results:**

For 65,803 adults (aged 18 to 59), the presence of multimorbidity (≥2) was associated with 32% higher odds of having severe tooth loss (95% CI, 1.17; 1.49) and 33% lower odds of having functional dentition (95% CI, 0.60; 0.75). For the 22,728 older adults (aged 60 and older), multimorbidity (≥2) was associated with a 17% higher odds of severe tooth loss (95% CI, 1.06; 1.29) and 23% lower odds of having functional dentition (95% CI 0.70; 0.85). The sensitivity analysis, excluding hypertension, confirmed our findings.

**Conclusions:**

Brazilian adults and older adults with multimorbidity are more likely to have severe tooth loss and less likely to have functional dentition.

**Supplementary Information:**

The online version contains supplementary material available at 10.1186/s12889-021-12392-2.

## Background

Tooth loss is an important public health concern for global health [1–3] that impacts peoples’ quality of life [4]. In addition, there is increasing evidence that tooth loss could be associated with diet-related chronic diseases [5] such as malnutrition [6], obesity [7], cardiovascular diseases [8], hypertension [9], diabetes [10], certain types of cancer [11, 12], and, ultimately, mortality [13].

The World Health Organization (WHO) defines 20 as the minimum number of permanent teeth required for individuals to take part in social activities and achieve an adequate masticatory function [14]. This number is known as a functional dentition [15]. However, severe tooth loss, defined by fewer than 10 remaining teeth in the oral cavity [16], could impact one’s quality of life in a more extensive way.

The oral microbiota may change with systemic diseases [17], which may contribute to tooth loss. Chronic diseases with increased inflammation are frequently linked to increased risk of periodontal disease [18], a stronger predictor of tooth loss. For example, rheumatoid arthritis is a systemic autoimmune disease characterised by chronic inflammation [19], leading to bone loss. Other chronic conditions such as dementia have already been associated with poorer oral health and increased tooth loss during the life course [20]. Traditionally, oral health research has focused on the impact of a single chronic condition on oral health. However, many people have multiple chronic conditions [21].

Multimorbidity is a significant health problem globally [22, 23], and it is increasing as a phenomenon of global ageing [23]. It is the simultaneous occurrence of health problems in the same person, usually operationalised by the occurrence of ≥2 or ≥ 3 chronic diseases [24, 25]. In the UK, one in four patients of primary health care users [26] and in the USA, 25% of the adult population, have multiple chronic conditions [27]. One large Brazilian study in older adults showed that multimorbidity was prevalent in 70% of individuals and was more frequent in women, poorer individuals, lower education level, aged 80 and over, non-white, and former smokers [24]. Another study has shown the dependent association of multimorbidity and edentulism later in life [28]. People who suffer from multiple chronic conditions are considered “heavy” users of health services [23] which in turn has a significant financial impact on societies globally. As multimorbidity and tooth loss share the same common individual risk factors [29], it is crucial to establish whether it is associated with tooth loss in large national samples, independently of the social determinants of health and contextual covariates in both adults and older adults. Such investigation is needed because of the potential adverse effects of chronic diseases on oral health and to implement public health interventions to address general and oral health conditions mutually. A previous investigation of the Brazilian National Health Survey in 2013 has shown that among individuals aged 18 or older 11% have lost all teeth and 23% did not have 20 remaining teeth [30].

Therefore, the main objective of the present investigation was to assess whether the presence of multimorbidity is associated with tooth loss, i.e. functional dentition and severe tooth loss, independently of individual and contextual covariates. We analysed data from a large nationally representative sample of Brazilian adults and older adults.

## Methods

The data analysed in this study came from the 2019 National Health Survey (*Pesquisa Nacional de Saúde* - PNS), designed to have a nationally representative sample of the Brazilian population. The 2019 PNS is a cross-sectional household survey with a sampling process carried out in three stages. Using clustering sampling techniques, each stage selection was conducted using probability proportional to size, in which first municipalities were selected, followed by census tracts and, finally, their households. More detailed information on PNS’s sampling procedures and inclusion criteria can be found elsewhere [31]. This study is based on participants who have answered the individual questionnaire, comprising 88,531 individuals aged 18 and older. The PNS data is freely available on the Brazilian Institute of Geography and Statistics (IBGE) website: https://www.ibge.gov.br/estatisticas/downloads-estatisticas.html

### Tooth loss assessment

Tooth loss was measured by asking: “Have you lost any of your permanent upper teeth?” Response options were 1) No; 2) Yes, I have lost (number) teeth; 3) Yes, I have lost all my upper teeth. The same question was asked for the lower permanent teeth. The 2019 Brazilian Health Survey considered complete dentition to be 32 teeth, 16 in the upper and 16 in the lower arch. Upper and lower self-reported tooth loss count was analysed classified into two levels of severity, regardless of the tooth position: 1) Functional dentition, defined as at least 20 permanent teeth present [32]; and 2) Severe tooth loss considered fewer than 10 remaining permanent teeth.

### Multimorbidity assessment

Chronic diseases were assessed with the following Yes/No questions: “Has a medical doctor ever told you that you have …?”. The chronic diseases investigated were: 1) hypertension, 2) diabetes, 3) depression, 4) back problems, 5) mental problems (schizophrenia, bipolar disorder, dementia and related), 6) asthma, 7) arthritis or rheumatism, 8) cancer, 9) heart problem, 10) stroke, 11) chronic obstructive pulmonary disease (COPD), 12) chronic kidney disease, and 13) work-related musculoskeletal disorder.

### Covariates

The following covariates included were considered at the individual and contextual levels: gender (men or women); age was divided into 18–34, 35–44 and 45–59 years for adults and 60–69, 70–79 and 80 years and over for older adults; level of education was classified into formative years (0 to 8) and formal education (8 or more years) for both adults and older adults. Officially named in Brazil as colour/race, the self-described race was assessed using five options according to the categories proposed by the Brazilian Institute of Geography and Statistics (IBGE): 1) white; 2) black; 3) yellow; 4) brown, and 5) indigenous. We further categorise this variable into white versus non-white. Self-reported smoking status was measured as those who never smoked and individuals who smoked in the past and/or are current smokers. The geographic region of residence (South, Southwest, Midwest, North, and Northwest) was the contextual covariate.

### Statistical analysis

Data analysis was performed using the Stata software version 14.2 (StataCorp LP, College Station, United States) using the survey module that considers the effects of stratification and conglomeration in estimating indicators and their precision measures. Absolute and relative frequencies, with their respective 95% confidence intervals (95% CI), were calculated for all variables. Age-standardised estimates for functional dentition and severe tooth loss (including edentulous) were reported for each covariate. We opted not to stratify edentulous at another category because severe tooth loss (fewer than 10 teeth present in the mouth) is a global health problem [16]. Associations between multimorbidity and levels of tooth loss were assessed through crude and adjusted logistic regressions and by reporting the Odds Ratio (OR) and their 95% confidence intervals. In addition, a sensitivity analysis excluding hypertension was performed. We followed the STROBE checklist for human observational studies [33]. All methods were performed in accordance with the relevant guidelines and regulations.

The Brazilian Committee approved the survey protocol on Ethics in Human Research (protocol number 3.529.376).

## Results

Out of the 90,846 selected households, the analytical sample comprised of 65,803 participants aged 18–59 and 22,728 older adults (aged 60 and older) who had information on all variables included in the study. Table 1 shows the sample characteristics. The age-standardised estimates of having a functional dentition were higher among white participants, men, earning above one minimum wage, higher level of education, non-smokers, and those with no multimorbidity, for both adults and older adults.

**Table 1:** Descriptive characteristics and proportions of Brazilian adults and older-adults and age-standardised prevalence of tooth loss

Variables	***n*** = 65,803	%	Presence of Functional dentition^**b**^	Severe tooth loss^**b**^		***n*** = 22,728	%	Presence of Functional dentition^**b**^	Severe tooth loss^**b**^
			% (95% CI)	% (95% CI)				% (95% CI)	% (95% CI)
			Adults					Older Adults	
**Multimorbidity**									
No (one or zero)	53,665	80.7	90.4 (90.0; 90.9)	5.0 (4.7; 5.4)		11,748	49.1	26.3 (24.3; 28.4)	61.5 (59.0; 63.9)
multimorbidity≥2	12,138	19.3	87.5 (86.7; 88.2)	6.5 (5.9; 7.0)		10,980	50.9	20.4 (18.7; 22.3)	68.7 (66.7; 70.7)
**Racial Groups**									
White	22,508	41.3	91.4 (90.8; 92.0)	4.7 (4.2; 5.2)		9901	50.5	27.8 (25.5; 30.1)	60.1 (57.3; 62.9)
Non-white^a^	43,295	58.7	88.0 (87.5; 88.5)	6.2 (5.9; 6.6)		12,827	49.5	18.6 (17.0; 20.3)	70.3 (68.2; 72.2)
**Sex**									
Female	34,334	52.2	87.8 (87.2; 88.4)	6.6 (6.2; 7.1)		12,535	56.7	20.5 (18.8; 22.3)	69.4 (67.3; 71.4)
Male	31,469	47.8	91.4 (90.9; 91.9)	4.3 (3.9; 4.6)		10,193	43.3	27.0 (25.3;28.7)	59.4 (57.1; 61.5)
**Per capita Income**									
< 1 minimum wage	38,046	53.8	85.4 (84.8; 85.9)	8.0 (7.6; 8.5)		10,250	41.7	14.5 (13.4; 15.6)	75.2 (73.5; 76.8)
≥ 1 minimum wage	27,738	46.2	93.3 (92.8; 93.7)	3.3 (3.0; 3.6)		12,475	58.2	29.7 (27.7; 31.7)	57.9 (55.3;60.3)
**Schooling years**									
< 8 years	25,689	34.9	83.8 (83.2; 84.4)	8.7 (8.2; 9.3)		16,414	70.1	16.6 (15.4; 18.0)	71.2 (69.1; 73.2)
≥ 8 years	40,114	65.1	95.0 (94.7; 95.2)	2.2 (1.9; 2.4)		6314	29.9	50.3 (47.2; 50.3)	38.9 (36.0; 41.9)
**Geographic region**									
North	13,610	8.3	87.3 (86.5; 88.2)	6.2 (5.6; 6.8)		3487	6.1	17.4 (15.4; 19.7)	68.3 (65.6; 70.8)
Northwest	22,966	26.8	85.2 (84.5; 85.8)	7.9 (7.4; 8.4)		7736	25.4	17.7 (16.1; 19.4)	71.2 (68.9; 73.4)
Midwest	7808	7.9	89.9 (89.0; 90.7)	5.6 (4.9; 6.4)		2373	6.4	23.0 (20.9; 25.2)	66.8 (64.3; 69.1)
Southwest	13,610	42.6	92.2 (91.5; 92.8)	4.2 (3.7; 4.8)		5825	46.4	30.0 (27.7; 32.4)	58.6 (56.0; 61.2)
South	7969	14.4	89.9 (89.1; 90.7)	4.9 (4.4; 5.5)		3307	15.7	26.7 (24.3;29.3)	58.0 (55.2; 60.8)
**Smoking status**									
yes/in the past	21,162	32.3	86.5 (85.9; 87.1)	7.4 (6.9; 7.9)		11,117	50.3	20.9 (19.4; 22.6)	65.8 (63.4; 67.3)
never	44,641	67.7	91.5 (91.0; 92.0)	4.2 (3.8; 4.7)		11,611	49.7	24.8 (23.1; 26.7)	65.4 (63.2; 68.3)
**Age Groups**									
**18–34**	24,115	40.8	99.4 (99.2; 99.5)	0.3 (0.2; 0.4)	**60–69**	24,247	56.3	45.3 (43.8; 46.8)	38.4 (36.9; 39.9)
**35–44**	18,033	25.8	95.3 (94.8; 95.8)	1.7 (1.5; 2.0)	**70–79**	13,209	30.1	28.0 (26.3; 29.8)	59.0 (57.0; 61.0)
**45–59**	23,655	33.4	74.4 (73.4; 75.4)	13.9 (13.1; 14.8)	**80 +**	6098	13.6	12.0 (9.9; 14.6)	78.5 (74.9; 81.7)

^a^Brown/Black/Indigenous/Asian

^b^Age-standardized

Table 2 displays the prevalence and weighted proportions for all self-reported chronic diseases. For older adults, the three most prevalent chronic conditions were hypertension (56%), back problems (31%) and diabetes (21%). For adults, the three most prevalent chronic conditions were back problems (19%), hypertension (17%) and depression (9,8%). Concerning diseases combinations, 20% of older adults have hypertension and back problems, and 7,4% had hypertension, back problems and arthritis. For adults, 5,2% had hypertension and back problems, and 1,5% had the combination of depression, back problems and hypertension.

**Table 2:** Prevalence and weighted proportions of morbidities in Brazilian adults and older adults. The National Health Survey, 2019

Adults (18–59 years)				Older-Adults (60 years and over)			
Multimorbidity	***n*** = 65,803	%	Associated morbidities	Morbidities	***n*** = 22,728	%	Associated morbidities
			mean				mean
**Back problems**				**Hypertension**			
yes	12,414	19.0	2.09	yes	12,428	56.0	2.35
**Hypertension**				**Back problems**			
yes	11,391	17.0	2.14	yes	6617	31.1	2.78
**Depression**				**Diabetes**			
yes	5876	9.8	2.64	yes	4305	20.3	2.83
**Mental problems (schizophrenia, bipolar disorder, dementia and related)**				**Arthritis or rheumatism**			
yes	3933	7.0	2.57	yes	4025	18.2	3.12
**Asthma**				**Heart problem**			
yes	3311	5.5	2.2	yes	2724	13	3.47
**Arthritis or rheumatism**				**Depression**			
yes	3174	4.6	2.92	yes	2366	11.8	3.59
**Diabetes**				**Cancer**			
yes	3053	4.7	2.53	yes	1420	6,7	3.14
**Heart problem**				**Stroke**			
yes	1987	3.1	3.04	yes	1271	5,6	3.47
**Work-related musculoskeletal disorder**				**Mental problems (schizophrenia, bipolar disorder, dementia and related)**			
yes	1350	2.6	2.73	yes	859	4,7	3.87
**Cancer**				**Asthma**			
yes	894	1.4	2.51	yes	983	4,6	3.58
**COPD: chronic obstructive pulmonary disease**				**COPD: chronic obstructive pulmonary disease**			
yes	671	1.3	2,93	yes	587	2,9	4
**chronic kidney disease**				**chronic kidney disease**			
yes	751	1.2	2.9	yes	529	2,6	3.92
**Stroke**				**work-related musculoskeletal disorder**			
yes	703	1.0	3.1	yes	360	2,2	3.72
**Main dyad combinations**				**Main dyad combinations**			
Back problems + Hypertension	3235	5.2	3.3	Hypertension + Back problems	4082	20.0	3.5
Back problems + Depression	2133	3.7	3.7	Hypertension + Diabetes	3169	14.8	3.7
Hypertension + Depression	1850	3.0	3.8	Back problems + Diabetes	1296	6.7	4.1
**Main Triad combinations**				**Main Triad combinations**			
Back problems + Hypertension+ Depression	823	1.5	4.6	Hypertension+ Back problems + Diabetes	1022	5.2	4.4
Back problems + Hypertension+ Mental problems	443	0.8	4.6	Hypertension+ Back problems + Arthritis	1426	7.4	4.3
Back problems + Hypertension+ Asma	287	0.5	4.9	Hypertension+ Back problems + Heart problem	840	4.5	4.7

Table 3 shows the odds ratios (OR) for older adults with multimorbidity (≥2 or ≥ 3) compared to their counterparts. We found that those with ≥two chronic diseases had a 23% (95% CI 0.70; 0.85) lower chance of having a functional dentition and 17% (95% CI 1.06;1.29) higher chance of having severe tooth loss (including edentulous), independently of individual and contextual covariates, important area-level characteristics in the country. We found similar results for multimorbidity ≥3. Our sensitivity analysis (Additional file 1: Appendix 1), excluding those individuals with hypertension, confirmed our findings and showed a similar pattern.

**Table 3:** Logistic regression coefficients for the association of tooth loss and multimorbidity in Brazilian older adults (*n* = 22,728). The Brazilian National Health Survey, 2019

Multimorbidity	Presence of Functional Dentition		Severe tooth Loss	
	Unadjusted	Adjusted^**a**^	Unadjusted	Adjusted^**a**^
	OR (95%CI)	OR (95%CI)	OR (95%CI)	OR (95%CI)
**no**	1	1	1	1
**multimorbidity ≥ 2**	0.68 (0.62; 0.72)	0.77 (0.70; 0.85)	1.37 (1.26; 1.49)	1.17 (1.06; 1.29)
**no**	1	1	1	1
**multimorbidity ≥ 3**	0.66 (0.60; 0.74)	0.76 (0.68; 0.86)	1.39 (1.26; 1.55)	1.18 (1.05; 1.32)

^a^Adjusted for sex, race, income, schooling, age groups, geographic region and smoking status

Table 4 showed the coefficients (OR) for adults with multimorbidity (≥2 or ≥ 3). We found that adults with multimorbidity ≥2 chronic diseases had a 33% (95% CI 0.60; 0.75) lower chance of having a functional dentition and a 32% (95% CI 1.17; 1.49) higher chance of severe tooth loss. We found similar results for multimorbidity ≥3. Our sensitivity analysis (Additional file 1: Appendix 2), excluding those individuals with hypertension, confirmed our main findings.

**Table 4:** Logistic regression coefficients for the association of levels of tooth loss and multimorbidity in Brazilian adults (*n* = 65,803). The Brazilian National Health Survey, 2019

Multimorbidity	Presence of Functional Dentition		Severe tooth Loss	
	Unadjusted	Adjusted^**a**^	Unadjusted	Adjusted^**a**^
	OR (95%CI)	OR (95%CI)	OR (95%CI)	OR (95%CI)
**no (0 or 1 morbidity)**	1	1	1	1
**multimorbidity ≥ 2**	0.34 (0.31; 0.38)	0.67 (0.60; 0.75)	2.80 (2.50; 3.11)	1.32 (1.17; 1.49)
**no (0 or 1 morbidity)**	1	1	1	1
**multimorbidity ≥ 3**	0.28 (0.24; 0.31)	0.61 (0.52; 0.70)	3.39 (2.96; 3.90	1.42 (1.22; 1.66)

^a^Adjusted for sex, race, income, schooling, age, geographic region and smoking status

## Discussion

The main finding of this large nationally representative investigation is that multimorbidity was associated with tooth loss in both Brazilian adults and older adults. Furthermore, individuals with multimorbidity were less likely to have a functional dentition and had a higher risk of having severe tooth loss, independently of sociodemographic, behavioural and contextual covariates, highlighting the importance of monitoring the oral health of individuals with multimorbidity.

Traditionally, oral health research has focused on a single disease and its association with tooth loss, such as diabetes [34], hypertension [9] and cancer [12]. However, many people have several chronic diseases. Tooth loss is a complex measure in oral health epidemiology [2], and its occurrence at earlier stages of life could predict future tooth loss and negligent attitudes toward general health, favouring the presence of multimorbidity. Our findings showed that 50% of older adults and 20% of adults have multimorbidity, showing its importance as a public health issue. The prevalence of multimorbidity found in the present study is similar to other studies in older adults in China (45%) and Ghana (48%) [35]. Another Brazilian study in older adults has shown a prevalence of 70% of individuals with multimorbidity [24]. The explanation for this higher prevalence could be attributed to the inclusion of eyes diseases such as cataracts and high cholesterol [24].

Chronic diseases may change the oral microbiota [17], which in turn may contribute to tooth loss. In addition, multiple chronic diseases with increased inflammation are frequently linked to increased risk of periodontal disease [18], a stronger predictor of tooth loss. Moreover, recent studies have shown plausible explanations on how the oral microbiota, especially periodontal disease, might play an important role in systemic health [36] and Alzheimer’s disease [37]. One previous investigation has shown a dependent association of multimorbidity and edentulism later in life [28], but the impact on functional dentition was not well established. During the life course, dementia has been associated to tooth loss through three different mechanisms: nutritional status, occlusal contacts and inflammation [20]. One possible explanation could be that chronic periodontitis could result in tooth loss, but not before the inflammation has affected the central nervous system, impairing cognition. However, one proposed explanation is that people with better childhood cognitive function have better oral health and access to routine dental care as they go through life, losing fewer teeth along the life course. They are also much more likely to have a better cognitive function later in life [20]. To the authors’ knowledge, this is the largest observational study using nationally representative data from Brazil to investigate the association of multimorbidity with tooth loss severity.

The adequate management of individuals with multimorbidity is a complex challenge and requires good planning and articulation of actions from the Brazilian Unified Health System (SUS) to meet this demand and special attention for oral health teams to prevent tooth loss in adults and older adults. In our investigation, multimorbidity was an independent associated factor with important levels of tooth loss (functional dentition and severe tooth loss) that are important indicators for public and global health. Addressing this issue will take more than the traditional approach, which radical reforms should accompany oral health systems and multidisciplinary teamwork [38]. An earlier diagnosis of multimorbidity (in adulthood) should be adopted since our investigation showed that adults with multimorbidity had higher chance of having tooth loss adjusted for the main social determinants of health [29]. The Brazilian Unified Health System should focus on promoting oral health behaviours such as toothbrushing twice or more times a day and the use of dental floss in these individuals. Most people with no multimorbidity (95.4%) brushed their teeth twice or more and 65% declared that they used dental floss compared to 93.3% of individuals with multimorbidity (≥2) who brushed their teeth twice or more and only 55% reported the use of dental floss. This difference between the groups was significant. Causal associations between amenable risk factors and non-communicable diseases (NCDs), a large proportion of the multimorbidity burden, are well established [21]. However, it is necessary to include oral health in this agenda, as it shares the same risk factors. Oral health has been neglected globally [39] and should be included in the political agenda [3], especially on upstream oral health policies like the sugar tax [39].

An increase in preventive dental check-ups could explain a large share in tooth loss between different racial groups in older adults [32] and could be a potential pathway to integrative care with other professionals in the Family Health Strategy program to address multimorbidity. Although the number of dentists in Brazil is high, they are not equally distributed across the five large geographic regions and across private and public sectors [40]. This could cause barriers to dental care access in the country, especially for those who need public services, i.e., the poorest individuals. In Brazil, between 2007 and 2014, there was no significant increase in the number of dentists working in general practice in the public sector [40]. On the other hand, in private practice, there was an average increase of 24.5%. This extensive discrepancy results in severe barriers to preventive dental services of the public sector users compromising the oral health of those who need it the most, i.e., the poorest, with the lowest level of education, non-white and individuals at higher risk for multimorbidity and tooth loss. For example, in 2013, data from the Brazilian National Health Survey have showed that, among individuals aged 18 or older, 11% were edentulous and 23% had fewer than 20 remaining teeth [30]. In the 2019 Survey, in the same age group, 10.3% were edentulous and 21.3% did not have 20 remaining teeth. The similar parameters of 2013 and 2019 could be associated by the limited access to the public health system as explained previously [40].

This study has some strengths and limitations that should be acknowledged. A key strength was the use of a nationally representative sample of Brazilian adults and older adults. The self-reported nature of tooth loss assessment may lead to information bias. Although clinical data regarding tooth loss might have strengthened our findings, previous research has shown a good concordance between self-reported tooth loss and clinical evaluation in national surveys [41]. Moreover, we used two definitions of tooth loss used internationally for comparison between populations. It was not also possible to establish whether tooth loss and occlusion, an essential predictor of maintaining masticatory functions and quality of life. The definition of multimorbidity is complex and vary across different studies and countries, and it is another limitation. However, we used two different classifications for multimorbidity (with the combination of 2 or 3 chronic diseases) and have conducted a sensitivity analysis to overcome this issue. For example, self-reported hypertension is a commonly used condition to measure multimorbidity [42]. Nevertheless, it is considered to be a risk factor for other non-communicable chronic diseases [43]. We, therefore, carried out an additional analysis, excluding hypertension, and confirmed our findings. We have also investigated the five Brazilian geographic regions to infer the place of residence, an important area-level characteristic. Our findings confirmed existing area-level differences in the country that require further investigation, especially considering other variables such as health services coverage, nutritional statuses, frailty and access to health services. Another limitation of our study is its cross-sectional design that may not allow to establish a temporal relationship between exposures and outcomes. Our findings clearly showed the association of presence of multimorbidity with functional dentition and severe tooth loss. However, tooth loss as a longitudinal observation was not tested. Specifying the role of potential pathways by which tooth loss-related mortality is mediated by systemic inflammation and nutritional status will potentially increase the importance of dental treatment for general health [44]. Finally, the lack of data on eyes diseases such as cataracts and high cholesterol, conditions commonly used in international multimorbidity studies [35], may have resulted in underestimating multimorbidity in the present study.

In conclusion, adults and older adults with multimorbidity are more likely to have severe tooth loss and less likely to have functional dentition. Therefore, our findings reinforce the importance of the Family Health Strategy of the Brazilian Unified Health Service to identify those individuals living with multimorbidity to tackle its adverse effects on oral health. Our study also highlights the importance of including oral health on the non-communicable diseases burden agenda.

## Supplementary Information

**Additional file 1: Appendix 1.** Logistic regression coefficients for the association of tooth loss and multimorbidity (excluding hypertension) in Brazilian older adults. The Brazilian National Health Survey, 2019. **Appendix 2.** Logistic regression coefficients for the association of levels of tooth loss and multimorbidity (excluding hypertension) in Brazilian adults. The Brazilian National Health Survey, 2019.

## Data Availability

The data that support the findings of this study are openly available in [Brazilian Institute of Geography and Statistics (IBGE)] at [https://www.ibge.gov.br/estatisticas/downloads-estatisticas.html].
