# Supplementary material for: Multimorbidity and tooth loss: the Brazilian National Health Survey, 2019
Source: BMC Public Health. 2021 Dec 20;21:2311. doi: 10.1186/s12889-021-12392-2 (PMC8691078; doi:10.1186/s12889-021-12392-2)
Supplement: Supplementary file 1 — Additional file 1: Appendix 1. Logistic regression coefficients for the association of tooth loss and multimorbidity (excluding hypertension) in Brazilian older adults. The Brazilian National Health Survey, 2019. Appendix 2. Logistic regression coefficients for the association of levels of tooth loss and multimorbidity (excluding hypertension) in Brazilian adults. The Brazilian National Health Survey, 2019. [file 12889_2021_12392_MOESM1_ESM.docx]

Appendix

| Appendix 1. Logistic regression coefficients for the association of tooth loss and multimorbidity (excluding hypertension) in Brazilian older adults. The Brazilian National Health Survey, 2019 | | | | | | | | |
| --- | --- | --- | --- | --- | --- | --- | --- | --- |
| **multimorbidity** | | **Presence of Functional Dentition** | | |  | **Severe tooth Loss** | | |
|  |  | **Unadjusted** |  | **Adjusted*** |  | **Unadjusted** |  | **Adjusted*** |
|  |  | **OR (95%CI)** |  | **OR (95%CI)** |  | **OR (95%CI)** |  | **OR (95%CI)** |
|  |  |  | | |  |  | | |
|  |  |  |  |  |  |  |  |  |
|  | **no** | 1 |  | 1 |  | 1 |  | 1 |
|  | **multimorbidity≥ 2** | 0.78 (0.70; 0.87) |  | 0.85 (0.75; 0.93) |  | 1.22 (1.11; 1.34) |  | 1.12 (1.00; 1.24) |
|  | **no** | 1 |  | 1 |  | 1 |  | 1 |
|  | **multimorbidity ≥ 3** | 0.79 (0.69; 0.90) |  | 0.84 (0.73; 0.98) |  | 1.17 (1.03; 1.32) |  | 1.06 (0.92; 1.22) |
| *Adjusted for sex, race, income, schooling, age groups, geographic region and smoking status. | | | | | | |  |  |

| Appendix 2. Logistic regression coefficients for the association of levels of tooth loss and multimorbidity (excluding hypertension) in Brazilian adults. The Brazilian National Health Survey, 2019 | | | | | | | | |
| --- | --- | --- | --- | --- | --- | --- | --- | --- |
| **multimorbidity** | | **Presence of Functional Dentition** | | |  | **Severe tooth Loss** | | |
|  |  | **Unadjusted** |  | **Adjusted*** |  | **Unadjusted** |  | **Adjusted*** |
|  |  | **OR (95%CI)** |  | **OR (95%CI)** |  | **OR (95%CI)** |  | **OR (95%CI)** |
|  |  |  | | |  |  | | |
|  |  |  |  |  |  |  |  |  |
|  | **no** | 1 |  | 1 |  | 1 |  | 1 |
|  | **multimorbidity≥ 2** | 0.40 (0.36; 0.44) |  | 0.70 (0.62; 0.78) |  | 2.41 (2.14; 2.71) |  | 1.29 (1.12; 1.47) |
|  | **no** | 1 |  | 1 |  | 1 |  | 1 |
|  | **multimorbidity ≥ 3** | 0.31 (0.27; 0.36) |  | 0.62 (0.52; 0.73) |  | 3.05 (2.58; 3.60) |  | 1.43 (1.19; 1.71) |
| *Adjusted for sex, race, income, schooling, age, geographic region and smoking status. | | | | |  |  |  |  |
